# Rationale and methods of the Antioxidant and NMDA receptor blocker Weans Anoxic brain damage of KorEa OHCA patients (AWAKE) trial

**DOI:** 10.1186/s13063-022-06452-0

**Published:** 2022-07-23

**Authors:** Jin-Ho Choi, Byeong Jo Chun, Seok Ran Yeom, Sung Phil Chung, Young Hwan Lee, Yun-Hee Kim, Ji Sung Lee, Jin Hwan Lee, Hwan Goo Lee, Jing Yu Jin, Chun San An, Byoung Joo Gwag

**Affiliations:** 1 Samsung Medical Center, Department of Emergency Medicine, Sungkyunkwan University School of Medicine, Seoul, Republic of Korea; 2 Chonnam National University Hospital, Department of Emergency Medicine, Chonnam National University Medical School, Gwangju, Republic of Korea; 3 Pusan National University Hospital, Department of Emergency Medicine, Pusan National University School of Medicine, Busan, Republic of Korea; 4 Gangnam Severance Hospital, Department of Emergency Medicine, Yonsei University College of Medicine, Seoul, Republic of Korea; 5 Department of Emergency Medicine, Soonchunhyang University Bucheon Hospital, Bucheon-si, Gyeonggi-do, Republic of Korea; 6 Samsung Medical Center, Department of Physical and Rehabilitation Medicine, Sungkyunkwan University School of Medicine, Seoul, Republic of Korea; 7 Department of Clinical Epidemiology and Biostatistics, Asan Medical Center, University of Ulsan College of Medicine, Seoul, Korea; 8 GNT Pharma Co. Ltd, Yongin-si, Gyeonggi-Do Republic of Korea

**Keywords:** Out-of-hospital cardiac arrest, Randomized controlled trial, Ischemic-reperfusion brain injury, Neu2000K, NMDA antagonist, Free radical scavenger

## Abstract

**Background:**

Ischemic brain injury is a major hurdle that limits the survival of resuscitated out-of-hospital cardiac arrest (OHCA).

**Methods:**

The aim of this study is to assess the feasibility and potential for reduction of ischemic brain injury in adult OHCA patients treated with high- or low-dose Neu2000K, a selective blocker of *N*-methyl-d-aspartate (NMDA) type 2B receptor and also a free radical scavenger, or given placebo. This study is a phase II, multicenter, randomized, double-blinded, prospective, intention-to-treat, placebo-controlled, three-armed, safety and efficacy clinical trial. This trial is a sponsor-initiated trial supported by GNT Pharma. Successfully resuscitated OHCA patients aged 19 to 80 years would be included. The primary outcome is blood neuron-specific enolase (NSE) level on the 3rd day. The secondary outcomes are safety, efficacy defined by study drug administration within 4 h in > 90% of participants, daily NSE up to 5th day, blood S100beta, brain MRI apparent diffusion coefficient imaging, cerebral performance category (CPC), and Modified Rankin Scale (mRS) at 5th, 14th, and 90th days. Assuming NSE of 42 ± 80 and 80 ± 80 μg/L in the treatment (high- and low-dose Neu2000K) and control arms with 80% power, a type 1 error rate of 5%, and a 28% of withdrawal prior to the endpoint, the required sample size is 150 patients.

**Discussion:**

The AWAKE trial explores a new multi-target neuroprotectant for the treatment of resuscitated OHCA patients.

**Trial registration:**

ClinicalTrials.gov NCT03651557. Registered on August 29, 2018.

## Administrative information

The items of checklist for clinical trial protocol recommended by Standard Protocol Items: Recommendations for Interventional Trials (SPIRIT) 2013 statement are indicated by braces (http://www.equator-network.org/reporting-guidelines/spirit-2013-statement-defining-standard-protocol-items-for-clinical-trials/).

Note: the numbers in curly brackets in this protocol refer to SPIRIT checklist item numbers. The order of the items has been modified to group similar items (see http://www.equator-network.org/reporting-guidelines/spirit-2013-statement-defining-standard-protocol-items-for-clinical-trials/).

**Table:** 

Title {1}	Rationale and methods of the Antioxidant and NMDA receptor blocker Weans Anoxic brain damage of KorEa OHCA patients (AWAKE) trial
Trial registration {2a and 2b}.	ClinicalTrials.gov Identifier: NCT03651557. Registered August 29, 2018 https://clinicaltrials.gov/ct2/show/NCT03651557
Protocol version {3}	Version 1.3 as of December 20, 2017
Funding {4}	This study is funded by GNT Pharma and a grant from the Ministry of Health and Welfare of the Republic of Korea (HI20C0410).
Author details {5a}	Jin-Ho Choi, MD, PhD; Samsung Medical Center, Department of Emergency Medicine, Sungkyunkwan University School of Medicine, Seoul, Republic of Korea Byeong Jo Chun, MD, PhD; Chonnam National University Hospital, Department of Emergency Medicine, Chonnam National University Medical School, Gwangju, Republic of Korea Seok Ran Yeom, MD, PhD; Pusan National University Hospital, Department of Emergency Medicine, Pusan National University School of Medicine, Busan, Republic of Korea Sung Phil Chung, MD, PhD; Gangnam Severance Hospital, Department of Emergency Medicine, Yonsei University College of Medicine, Seoul, Republic of Korea Young Hwan Lee, MD, PhD; Soonchunhyang University Bucheon Hospital, Department of Emergency Medicine, Bucheon-si, Gyeonggi-do, Republic of Korea Yun-Hee Kim, MD, PhD; Samsung Medical Center, Department of Physical and Rehabilitation Medicine, Sungkyunkwan University School of Medicine, Seoul, Republic of Korea Ji Sung Lee, PhD; Department of Clinical Epidemiology and Biostatistics, Asan Medical Center, University of Ulsan College of Medicine, Seoul, Korea Jin Hwan Lee, PhD, Hwan Goo Lee, MS, Jing Yu Jin, PharmD, PhD, Chun San An, MD, PhD, and Byoung Joo Gwag, PhD; GNT Pharma Co. Ltd., Yongin-si, Gyeonggi-Do, Republic of Korea
Name and contact information for the trial sponsor {5b}	Byoung Joo Gwag, PhD; GNT Pharma Co. Ltd., 23, Yonggu-daero 1855 beon-gil, Giheung-gu, Yongin-si, Gyeonggi-Do, Republic of Korea Email: bjgwag@gntpharma.com
Role of sponsor {5c}	The sponsors have no role in the study design, data collection, management, analysis, and interpretation of data. The sponsors are not involved in the writing of the report and the decision to submit the report.

## Introduction

### Background and rationale {6a}

Out-of-hospital cardiac arrest (OHCA) is a major public health challenge. The global incidence of OHCA ranges from 20 to 140 per 100,000 person-years, with low short-term survival rates from 2 to 16% [1–4]. Global brain ischemia and reperfusion are the key pathophysiology underlying OHCA. Manual chest compression attempts during resuscitation typically provide only 15 to 25% of normal cardiac output [5]. Impaired cerebral perfusion does not allow the delivery of vital materials such as oxygen and glucose, leading to the depletion of metabolic energy. In addition, full restoration of cerebral perfusion is not completely harmless and causes additional reperfusion injuries [6].

Excitotoxicity is one of the major pathogenic mechanisms underlying ischemic and reperfusion brain injury [7]. It is predominantly triggered by the overactivation of *N*-methyl-d-aspartate (NMDA) receptors and followed by extracellular calcium influx, loss of cell membrane potential, oxidative stress, and eventually, neuronal death [8, 9]. Toxic free radicals such as superoxide and hydrogen peroxide are produced and accumulated in the neurons and glia through mechanisms involving reoxygenation and iron overload during reperfusion as well as intracellular calcium overload subsequent to the activation of NMDA receptors, which triggers delayed neuronal and glial cell death [10, 11].

Given the pivotal role of NMDA receptors in excitotoxicity, NMDA receptor antagonists have been considered as one of the promising therapeutic targets for neuroprotection against ischemic and reperfusion neuronal injury [12]. However, despite the initial encouraging results in preclinical studies, prior NMDA receptor blockers have failed to be translated for clinical use [13, 14]. The explanation for these failures of translation is likely to be multifactorial. The blockade of excitotoxicity prevents rapidly evolving neuronal death after hypoxic-ischemic brain injury; however, it delays neuronal apoptosis through mechanisms involving intracellular Ca^2+^ ion deficiency and caspase-3 activation [7, 15–19]. The clinical use of NMDA receptor antagonists is also limited by the short therapeutic time window and adverse effects such as nausea, vomiting, cardiovascular and psychomimetic effects, and oxidative stress that causes delayed brain damage after ischemic reperfusion injury [20–22].

These therapeutic boundaries may be overcome by understanding the pathophysiology first and then improving the therapeutic strategies subsequently. Targeted temperature management, which significantly reduces excitatory amino acid neurotransmitters that activate NMDA receptors, has been widely performed for post-resuscitation care of OHCA [23–25]. NMDA receptor has dual roles in neuronal survival and death. The stimulation of extrasynaptic NMDA receptors is associated with pro-death pathways, whereas the stimulation of synaptic NMDA receptors activates pro-survival signaling pathways and enhances the intrinsic antioxidant system [26, 27]. Therefore, selective NMDA receptor blockade focusing on the neuroprotective role without causing excessive reduction of calcium influx and boosting the antioxidative defenses is expected to improve the neurological outcome of OHCA [7, 11, 18, 19].

**Fig. 1:**
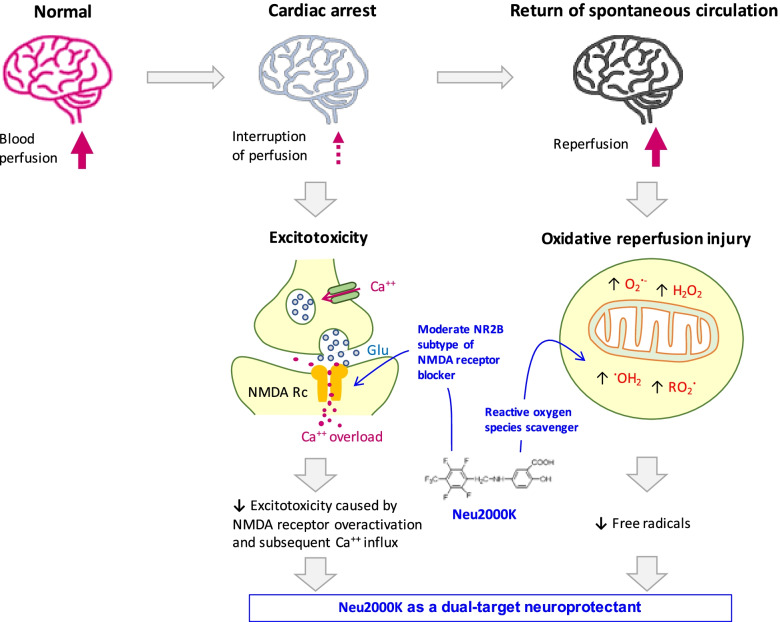
Mechanism of Neu2000K as a dual-target neuroprotectant for ischemic and reperfusion brain injury. In patients suffering from cardiac arrest, oxygen and glucose are deprived from the brain, which results in glutamate release and accumulation at the synaptic cleft. Excess glutamate causes acute and fulminant neuronal death through the overactivation of calcium ion-permeable NMDA receptors. Free radicals such as superoxide and hydrogen peroxide are produced in the mitochondria over hours and days after the return of spontaneous circulation. Such prolonged and excessive oxidative stress causes delayed cell death including the neurons and glia. Neu2000K moderately and selectively blocks the NR2B subtype of NMDA receptor, which activates pro-survival signaling pathways without causing excessive calcium influx, which can reduce acute neuronal death. Neu2000K also functions as a potent reactive oxygen species scavenger and reduces oxidative stress or reperfusion injury, which can reduce delayed brain cell death

Neu2000 (nelonemdaz, an international nonproprietary name, 2-hydroxy-5-[2,3,5,6-tetrafluoro-4-trifluoromethyl-benzylamino)-benzoic acid]) is a novel, multi-target neuroprotective agent. Neu2000 was designed to prevent NMDA receptor-mediated excitotoxicity by moderately and selectively inhibiting the NR2B subtype of the NMDA receptor and also to block oxidative stress as a potent free radical scavenger. It remarkably attenuates neuronal death, oxidative stress, and infarct after ischemic-reperfusion brain injury in animal models; however, it does not cause neurotoxicity unlike the other potent NMDA receptor antagonists (Fig. 1) [22]. Compared to NMDA antagonists or antioxidants alone, the administration of Neu2000 showed better neuroprotection and a longer therapeutic window [22, 28–30]. In two phase I studies of Neu2000K (Neu2000 potassium salt) for 165 healthy subjects conducted in the USA and China, the intravenous administration of even 6000 mg Neu2000K did not produce serious adverse events such as psychosis [11]. Two phase II studies of Neu2000K for acute ischemic stroke patients receiving recanalization therapy have been completed (NCT04486430, NCT02831088). Based upon promising safety and efficacy profiles in preclinical and clinical studies, a phase III study of Neu2000K has been initiated for acute ischemic stroke patients receiving endovascular thrombectomy (NCT05041010).

The present trial will investigate the neuroprotective capabilities of Neu2000K for patients resuscitated from OHCA and undergoing targeted temperature management.

### Objectives {7}

The primary objective of this trial is to evaluate the feasibility and potential for reduction of ischemic brain injury in adult OHCA patients treated with high- or low-dose Neu2000K, and the second objective is to evaluate the safety of intravenous administration of Neu2000K in adult OHCA patients.

### Trial design {8}

The Antioxidant and NMDA receptor blocker Weans Anoxic brain damage of KorEa OHCA patients (AWAKE) trial is a sponsor-initiated, randomized, placebo-controlled, double-blinded, prospective, intention-to-treat, three-armed, safety and efficacy clinical phase II trial.

## Methods: participants, interventions, and outcomes

### Study setting {9}

Patients will be enrolled in the following tertiary university hospitals offering highly specialized cardiac care in Korea: Samsung Medical Center, Chonnam National University Hospital, Pusan National University Hospital, Gangnam Severance Hospital, and Soonchunhyang University Bucheon Hospital.

GNT Pharma and the Ministry of Health and Welfare of the Republic of Korea are sponsors of this trial. The trial will be conducted with the help of a clinical research organization, Medical Excellence based in Seoul. The authors are solely responsible for the design and conduct of this study, all study analyses, the drafting and editing of the paper, and its final contents.

### Eligibility criteria {10}

#### Inclusion criteria

Adult patients aged 19–80 years, with a witnessed OHCA, successful resuscitation defined by the sustained return of spontaneous circulation (ROSC) time ≥ 20 min, and planned or initiated targeted temperature management (32–34 C for 24 h), are eligible for inclusion in this study. These criteria are designed to identify patients with presumed cardiac causes. Intravenous administration of the investigational drug in these patients should be feasible within 4 h after ROSC.

#### Exclusion criteria

The following are the exclusion criteria: unwitnessed arrest, resuscitation time ≥ 60 min, no indication for targeted temperature management, simultaneous application of ECMO during resuscitation, respiratory arrest without concomitant or ensuing cardiac arrest, suspected or confirmed intracranial hemorrhage, major stroke, significant baseline neurological disease, arrest subsequent to traumatic injury or major bleeding, end-stage malignancy or non-cardiac organ dysfunction, very poor expected neurological or medical prognosis, no confirmed absence of pregnancy in a potentially fertile woman, and hypersensitivity to aspirin or sulfasalazine. Exclusion criteria are considered only if they are known before the start of treatment.

### Who will take informed consent? {26a}

Eligible subjects are unconscious and incapacitated to give informed consent. Therefore, written informed consent is obtained from an immediate family member who serves as a legal representative prior to the enrollment. Consent will be obtained by the investigator or co-investigators.

### Additional consent provisions for collection and use of participant data and biological specimens {26b}

This trial involves collecting biological specimens to be analyzed. On the consent form, participants will be asked if they agree to the use of their data should they choose to withdraw from the trial. Participants will also be asked for permission for the research team to share relevant data with researcher or regulatory authorities, where relevant.

## Interventions

### Explanation for the choice of comparators {6b}

**Fig. 2:**
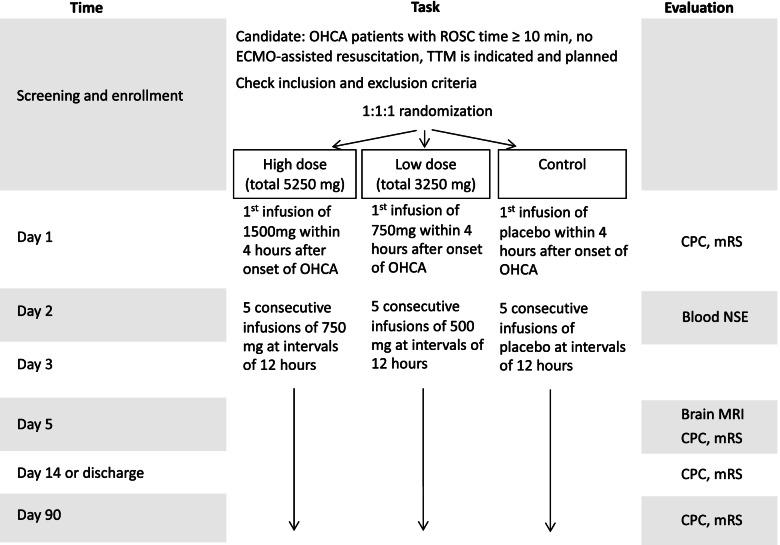
Study flow. OHCA, out-of-hospital cardiac arrest; ROSC, return of spontaneous circulation; ECMO, extracorporeal membranous oxygenation; TTM, targeted temperature management; CT, computed tomography; NSE, neuron-specific enolase; MRI, magnetic resonance imaging; CPC, cerebral performance category; mRS, Modified Rankin Scale

The overview of the study flow is shown in Fig. 2. OHCA patients who arrived at the emergency department are considered to be enrolled in this study if ROSC is sustained and targeted temperature management is indicated or planned. The resuscitation team leader assesses eligibility for study participation. Informed consent is obtained from an immediate family member if all the inclusion criteria and no exclusion criteria are met accordingly. Aiming for the reduction of excitotoxicity using NMDA receptor antagonist, we have chosen to give a total of Neu2000 5250 mg 3250 mg or placebo for 3 days, as this is the dosage decided from prior phase I and phase II studies. Patients are randomly assigned to Neu2000K high dose, Neu2000K low dose, or placebo in a 1:1:1 ratio via a cloud-based randomization algorithm.

### Intervention description {11a}

#### Neu2000K high-dose group

The 1st infusion of 1500 mg within 4 h after OHCA onset, followed by 5 consecutive infusions of 750 mg at intervals of 12 h. The total dosage is 5250 mg.

#### Neu2000K low-dose group

The 1st infusion of 750 mg within 4 h after OHCA onset, followed by 5 consecutive infusions of 500 mg at intervals of 12 h. The total dosage is 3250 mg.

#### Placebo group

The 1st infusion of the same volume of saline within 4 h after OHCA onset, followed by 5 consecutive infusions of the same volume of saline at intervals of 12 h.

##### Post-resuscitation care

Post-resuscitation care including targeted temperature management for 24 h and optimizing physiological parameters is performed according to the clinical guidelines and local protocols.

### Criteria for discontinuing or modifying allocated interventions {11b}

The subject or the subject’s next of kin has the right to withdraw from the study at any time for any reason without prejudice to his or her future medical care. Any subject who withdraws consent to participate in the study will be removed immediately from the further treatment and/or study observation.

The investigator and the sponsor have the right to withdraw a subject from the study if any of the following occurs: unexpected significant intercurrent illness, refusal to continue clinical observations, or decision by the investigator that study termination is the best medical interest of the subject.

Unless specific request by the subject, the subject will be followed for the primary endpoint until the end of the study period. When a subject or legally acceptable representative request withdrawal of the study, the end of study case report form will be completed with an explanation for the withdrawal as well as a complete final evaluation and assessments. If the withdrawal of a subject is due to an adverse event, follow-up visits should be scheduled until the adverse event has been resolved or stabilized. Unless consent has been withdrawn, follow-up data on deaths and hospitalizations will be collected until study termination since the enrollment of the subject.

### Strategies to improve adherence to interventions {11c}

The medical professionals who are involved in the care of patients participating in the study have been trained in the study-specific procedures. A study flow sheet has been developed for study-specific interventions. To facilitate study and avoid confusion in the preparation of study drugs or storage of study samples, a validated and dedicated thermostat storage and a deep freezer have been installed in the clinical study room in the emergency department.

### Relevant concomitant care permitted or prohibited during the trial {11d}

There is no specific restriction on the standard of care for OHCA according to the contemporary guidelines. Patients will receive ventilator care, targeted temperature management of 36 °C for at least 24 h, sedation, and/or vasopressors and/or inotropes if needed at the discretion of the corresponding physician. Major cardiovascular interventions will not be delayed by the study intervention. Efforts will be made to maintain study drug infusion during specific treatment.

### Provisions for post-trial care {30}

Participating subjects will be insured by the health system responsible for the study site.

### Outcomes {12}

The primary endpoint is biomarker of cerebral injury assessed by blood NSE level on the 3rd day.

The secondary endpoints include the following: biomarkers of cerebral injury assessed by NSE on the 4th day, difference in blood NSE on the 1st and 3rd day, difference in blood NSE on the 4th and 5th day, and blood S100beta on the 4th day. Imaging marker of cerebral injury is assessed by brain MRI apparent diffusion coefficient (ADC) images on the 4th or 5th day. Clinical and functional markers of cerebral injury are assessed by cerebral performance category (CPC) and Modified Rankin Scale (mRS) on the 5th day, day 14, or at discharge within days 14 and 90. Safety is assessed by any adverse event during the study period.

### Participant timeline {13}

The study flow and participant timeline are shown in Fig. 2.

### Sample size {14}

The primary hypothesis of this trial is that patients receiving Neu2000K will have lower blood NSE levels on the 3rd day compared with those receiving placebo. Based on a prior study, the NSE level on the 3rd day of the control group was estimated to be about a mean of 90 μg/L and a standard deviation of 80 μg/L [31]. We assumed that any dose group compared to the placebo group can lower the NSE level on the 3rd day to 40 μg/L or less. Consequently, a total of 150 patients (50 per arm) are needed for this trial with the consideration of power of 84%, one-sided alpha of 0.05, and dropout rates of 28%.

### Recruitment {15}

The time required for recruiting the intended 150 patients is estimated to be 3 years from enrollment of the first patient. This estimate is based on the volume of OHCA patients in the participating centers.

## Assignment of interventions: allocation

### Sequence generation {16a}

Subjects who underwent screening successfully will be randomized into the study by the investigator or assigned co-investigator. To ensure balanced distribution among three treatment groups, subjects will be assigned to one of three treatment groups using site-specific stratified block randomization with a 1:1:1 ratio.

### Concealment mechanism {16b}

Before initiation of enrollment, pre-fabricated statistically validated allocation sequence sheets and investigational drugs labeled with the site-specific serial number will be provided to each site. Investigators in each site administer investigational drugs labeled with the corresponding serial number to the enrolled subjects.

Physicians and study coordinators will be blinded to the allocation sequence all throughout the trial. Assignment will be concealed until finishing the decision for the assessment of all participants and case report form database lock. Unblinding should only be allowed in emergency situation where the knowledge of the treatment is essential for the clinical decision of the subject or requirement for safety reporting to regulatory authorities.

### Implementation {16c}

The allocation sequence will be generated by those who are otherwise not involved in the trial conduction. Investigator and co-investigators in each site enroll the subject. An independent co-investigator assigns the study subject to treatment arms. Investigational drugs are prepared by the investigational product (IP)-preparing personnel and administered to the subject by the other co-investigators.

## Assignment of interventions: blinding

### Who will be blinded {17a}

All study investigators and participants are blinded to treatment allocation except the investigational product (IP)-preparing personnel. The randomization schedule is not accessible except for safety reasons, until the completion of this trial. Investigational drugs and the placebo are provided in an identical package. Treatment assignment will remain blind to both the patient and the treating physician except for IP-preparing personnel. The IP-preparing personnel are not involved in any other aspect of the study. Study outcomes will be assessed using standardized forms and procedures by separate certified investigators who are blinded to the treatment. The blinded-endpoint monitoring board will determine the primary endpoint through central adjudication. Brain MRI data will also be assessed by the imaging laboratory team who are unaware of the treatment allocation.

### Procedure for unblinding if needed {17b}

Emergency unblinding is allowed under the discretion of the lead study investigators, in such circumstances, which is automatically will be logged by the case report form system. Unblinding should only be allowed when the knowledge of the treatment is essential for the further management of the subject or if needed for safety reporting to regulatory authorities.

## Data collection and management

### Plans for assessment and collection of outcomes {18a}

All of patient data will be kept according to local practice and national legislation. The database will be maintained for 5 years and anonymized if requested by relevant authorities. Data from the analysis of biomarkers from the biobank will be stored on an approved server with a back-up function.

### Plans to promote participant retention and complete follow-up {18b}

The majority of patients are expected to remain in the intensive care unit during the initial 3 days and hospitalized at least 5 days, where most data collection and all biomarker sampling are scheduled. The contact information for patients and their next of kin is stored with the intention of inviting patients to the functional assessment at day 14 and day 90.

If the patient cannot visit the hospital, a follow-up questionnaire by letter, phone, or electrical message would be used for communication.

### Data management {19}

See the “Plans for assessment and collection of outcomes {18a}” section.

### Confidentiality {27}

All data will be kept in accordance with the approved data handling plan. Data from ordinary medical records will be entered into the dedicated secure database operated by Clinical Research Organization. Data will only be shared on reasonable requests with approval from the relevant authorities.

### Plans for collection, laboratory evaluation, and storage of biological specimens for genetic or molecular analysis in this trial/future use {33}

Genetic or molecular analyses are not planned and will be not performed.

## Statistical methods

### Statistical methods for primary and secondary outcomes {20a}

The data will be analyzed primarily on an intention-to-treat basis, which consists of patients who met both the inclusion and exclusion criteria, with informed consent from an immediate family member, and who are randomly allocated into one of three study arms. Data will be analyzed primarily on a full analysis set, which consists of patients who are randomly allocated into one of three study arms, and completed the test of blood NSE on the 3rd day. Statistical testing for the primary endpoint will be performed using Student’s *t*-test or Mann–Whitney’s *U*-test under a one-sided 5% significance level. Statistical reporting includes not only *p*-values but also confidence intervals. Secondary analyses will be performed using the chi-squared test, two-way analysis of variance test, and Cochran-Mantel–Haenszel test.

### Interim analyses {21b}

An interim analysis is not planned. The investigators will closely monitor the general safety of the trial participants.

### Methods for additional analyses (e.g., subgroup analyses) {20b}

In case of significant differences, comparisons between the placebo and Neu2000K low-dose groups, and placebo and Neu2000K high-dose groups will be conducted. For secondary endpoint analyses, Student’s *t*-test, Mann–Whitney’s *U*-test, chi-square test, Fisher’s exact test, and Cochran-Mantel–Haenszel shift test according to the type of variable will be used to compare the treatment groups. Secondary endpoints for statistical tests will be performed under a two-sided 5% significance level. Adjustment for multiple tests is not considered.

### Methods in analysis to handle protocol non-adherence and any statistical methods to handle missing data {20c}

Due to the extremely high early mortality of OHCA, the per-protocol analysis will consist of patients who received more than 80% of the planned study drug administration (5 or 6 infusions of the study drug or placebo). Any alternative method for missing values will not be used.

### Plans to give access to the full protocol, participant-level data, and statistical code {31c}

The study protocol, statistical code, and data can be supplied on a reasonable request and with approval from relevant authorities. Patient-level data can only be requested after full publication.

## Oversight and monitoring

### Composition of the coordinating center and trial steering committee {5d}

The investigators and sponsors supervise the AWAKE trial, which is a sponsor-initiated multi-center study. The steering committee includes investigators in each participating site, sponsor, and Clinical Research Organization (CRO).

### Composition of the data monitoring committee, its role, and reporting structure {21a}

Medical Excellence, a professional contract research organization, will monitor the study data, trial enrollment, and conduct. The data will be stored in a secure case report system. Hence, the size of this study is limited, no data monitoring committee will be formed. Safety reports will be filed to the local ethical committee and the Ministry of Food and Drug Safety of Korea.

### Adverse event reporting and harms {22}

Adverse events (AE) will be assessed daily for the first 5 days, and adverse events occurring after day 7 will be evaluated during follow-up on day 14 and day 90. At each assessment of adverse events, serious adverse events (SAE) and suspected unexpected serious adverse reactions (SUSAR) should be recorded in AE form by the investigator and evaluated. Record of each SAE and SUSAR requires the following variables: description of the event, onset and end of the event, severity, relation to the intervention, action taken, and outcome. Any adverse events occurring during the study will be treated according to the established standards, and the subject will be followed until the event has disappeared or stabilized. On a yearly basis, a safety report containing information on SAE and SUSAR will be submitted to the Ministry of Food and Drug Safety of Korea. For each AE, the investigator assesses potential causality between investigational products, and whether the reaction is suspected will be assessed by the sponsor. An SAE will be defined as any AE that results in death, is life-threatening, requires prolongation of hospitalization, or results in significant disability, including congenital anomaly or birth defect. The sponsor will be responsible to report all life-threatening lethal SUSARS to the Ministry of Food and Drug Safety of Korea as soon as possible and no later than 7 days after the event, and non-life-threatening SUSARs no later than 15 days after the event.

### Frequency and plans for auditing trial conduct {23}

The trial will be externally monitored by the Good Clinical Practice (GCP) unit at Samsung Medical Center and the Clinical Research Associate of Medical Excellence. There will be mandatory monitoring before and after the study and at least once during the study. The GCP will monitor the inclusion and exclusion criteria, consent obtained in all subjects according to legislation, and data included in the eCRF. The principal investigator will be responsible for all data in the eCRFs.

### Plans for communicating important protocol amendments to relevant parties (e.g., trial participants, ethical committees) {25}

Major modifications of protocol and changes to patient information will be implemented after approval from regulating authorities. Any deviations from the protocol will be fully documented using a breach report form.

### Dissemination plans {31a}

When completed, the study results will be submitted for publication in international peer-reviewed journals and presented at international conferences. The major results would be publicly available.

## Discussion

Ischemic neuronal injury is the major cause that limits the neurologically favorable survival of OHCA patients. Despite vast advances in resuscitation medicine, there is no currently approved drug targeting the reduction of ischemic reperfusion brain injury or improving the clinical outcome of OHCA. At present, there are four other trials enrolling OHCA patients with investigational drugs including 2-iminobiotin (a selective neuronal and inducible nitric oxide synthase inhibitor), steroid (an adrenal cortex hormone and also a suppressor of inflammation), tocilizumab (an interleukin-6 receptor antibody and modulates systemic inflammatory response), and vasopressin (a vasopressor) in the Netherlands, Denmark, and Korea (NCT02836340, NCT04624776, NCT03863015, NCT03191240) [32]. This trial investigates Neu2000K, a first-in-class multi-target neuroprotectant that moderately and selectively blocks the NR2B subtype of NMDA receptor and also functions as a potent reactive oxygen species scavenger. The AWAKE trial will provide valuable safety and efficacy data that documents the neuroprotective properties of Neu2000K in OHCA and provides a broader implementation and a more extensive phase III clinical trial.

### Trial status

This report describes the study protocol version 1.3 as of December 20, 2017. The first patient was randomized on November 29, 2018. As of May 15, 2022, 89 patients were recruited for this study. AWAKE is currently recruiting study patients at all participating centers.

## Data Availability

The study data can be supplied on a reasonable request and with approval from relevant authorities.

## Abbreviations

AWAKE: Antioxidant and NMDA receptor blocker Weans Anoxic brain damage of KorEa OHCA patients
CPC: Cerebral performance category
ECMO: Extracorporeal membranous oxygenation
NMDA: *N*-Methyl-d-aspartate
mRS: Modified Rankin Scale
OHCA: Out-of-hospital cardiac arrest
ROSC: Return of spontaneous circulation
